# Recent advances in understanding and managing chronic constipation

**DOI:** 10.12688/f1000research.15900.1

**Published:** 2018-10-15

**Authors:** David O. Prichard, Adil E. Bharucha

**Affiliations:** 1 Division of Gastroenterology and Hepatology, Mayo Clinic, 200 1st Street SW, Rochester, MN 55905, USA; 2 Clinical Enteric Neuroscience Translational and Epidemiological Research Program and Division of Gastroenterology and Hepatology, Mayo Clinic, 200 1st Street SW, Rochester, MN 55905, USA

**Keywords:** Constipation, diagnosis, management, lubiprostone, linaclotide, plecanatide, prucalopride, velusetrag, elobixibat, NGM282, tenapanor

## Abstract

Constipation, a condition characterized by heterogeneous symptoms, is common in Western society. It is associated with reduced physical health, mental health, and social functioning. Because constipation is rarely due to a life-threatening disease (for example, colon cancer), current guidelines recommend empiric therapy. Limited surveys suggest that fewer than half of treated individuals are satisfied with treatment, perhaps because the efficacy of drugs is limited, they are associated with undesirable side effects, or they may not target the underlying pathophysiology. For example, although a substantial proportion of constipated patients have a defecatory disorder that is more appropriately treated with pelvic floor biofeedback therapy than with laxatives, virtually no pharmacological trials formally assessed for anorectal dysfunction. Recent advances in investigational tools have improved our understanding of the physiology and pathophysiology of colonic and defecatory functions. In particular, colonic and anorectal high-resolution manometry are now available. High-resolution anorectal manometry, which is increasingly used in clinical practice, at least in the United States, provides a refined assessment of anorectal pressures and may uncover structural abnormalities. Advances in our understanding of colonic molecular physiology have led to the development of new therapeutic agents (such as secretagogues, pro-kinetics, inhibitors of bile acid transporters and ion exchangers). However, because clinical trials compare these newer agents with placebo, their efficacy relative to traditional laxatives is unknown. This article reviews these physiologic, diagnostic, and therapeutic advances and focuses particularly on newer therapeutic agents.

## Background

Symptoms of constipation are reported by 10% to 20% of adults worldwide ^1^. Classically, the term “constipation” refers to infrequent bowel motions or hard feces. However, the disorder is heterogeneous; patients report a variety of symptoms including reduced bowel motion frequency, straining, hard stools, the sensation of incomplete emptying, the sensation of anal blockage, or the use of digitation or positioning to aid defecation ( Box 1) ^2^. In affected individuals, physical health, mental health, and social functioning are reduced ^3, 4^. Despite this, only one-fifth of constipated individuals seek health-care advice ^5^. Yet, given the high prevalence of this condition, this represents over 8 million health-care visits ^6^ and $230 million in costs ^7^ annually in the United States.

**Table:** Box 1. Diagnostic criteria for functional constipation

**Two or more of the following must be present** I. Straining during more than one-fourth (25%) of defecations II. Lumpy or hard stools (Bristol Stool Form Scale 1–2) in more than one-fourth (25%) of defecations III. Sensation of incomplete evacuation in more than one-fourth (25%) of defecations IV. Sensation of anorectal obstruction/blockage in more than one-fourth (25%) of defecations V. Manual maneuvers to facilitate more than one-fourth (25%) of defecations (for example, digital evacuation and support of the pelvic floor) VI. Fewer than three spontaneous bowel movements per week These criteria must be fulfilled for the previous 3 months. The symptom onset must be at least 6 months prior to diagnosis.
**In addition** I. Loose stools are rarely present without the use of laxatives II. There are insufficient criteria for irritable bowel syndrome

Based upon Mearin *et al*. ^2^.

In the absence of alarm features, constipation is seldom due to a life-threatening organic disorder (for example, colon cancer) or another disease (for example, hypothyroidism). Hence, guidelines recommend initial empiric therapy for constipated patients rather than investigating for a cause (for example, with colonoscopy) ^8, 9^. Likewise, although primary constipation generally results from slow colonic transit, impaired rectal evacuation, or both ^8^, tests to evaluate these processes are recommended only in patients who do not respond to laxatives.

Slow colonic transit is associated with hard stools ^10^. Consequently, osmotic and stimulant laxatives are the two most commonly prescribed agents ^11, 12^. Osmotic laxatives (for example, polyethylene glycol or lactulose) retain water in the intestinal lumen, accelerating colonic transit and reducing the consistency of evacuated stool. Stimulant laxatives (for example, senna, bisacodyl, or glycerin) stimulate colonic contractions and the urge to defecate. Although laxatives increase bowel motion frequency ^13^, satisfaction is variable ^14^. In an internet-based survey of 1,355 patients with self-reported constipation in 10 European countries in 2009, 855 patients were taking laxatives. Of these patients, 28% were very satisfied or satisfied with their treatment, 44% were neutral, and 28% were dissatisfied with therapy. In particular, the symptom of bloating persists ^15^. Conceptually, satisfaction may be suboptimal because the efficacy of drugs is limited, they are associated with undesirable side effects, or they may not target the underlying pathophysiology. In particular, a substantial proportion of constipated patients have a defecatory disorder that may be associated with normal or slow colonic transit. Defecatory disorders are more appropriately treated with pelvic floor biofeedback therapy than with laxatives ^16– 18^.

## Advances in understanding the molecular pathophysiology of constipation

Abnormalities of ion channels within the intestine have been shown to affect secretion, absorption, motility, and sensation, potentially resulting in constipation, diarrhea, and irritable bowel syndrome (IBS) ^19^. The presence of these dysfunctional channels may be suggested by a family history of a functional bowel disorder ^19^. Documented “channelopathies” include those affecting the voltage-gated sodium channel Na _V_1.5, present on smooth muscle cells; the voltage-gated sodium channels Na _V_1.7 and Na _V_1.9, present on neurons; and ion exchange channels, present on enterocytes ^19^. Altered ion channel expression or function occurs because of genetic mutations, post-translational modification, or accessory protein malfunction. In particular, the voltage-gated sodium channel Na _V_1.5 has been associated with constipation ^20^. In one study, mutations, predominantly resulting in loss of function, were found in 7% of patients (4 out of 59) with constipation-predominant IBS (IBS-C) ^20^. Further studies are necessary to determine whether these mutations were responsible for the bowel symptoms.

In selected studies, about one in four patients with diarrhea-predominant IBS has high concentrations of fecal bile acids ^21^. Conversely, a small fraction (that is, about 6%) of patients with IBS-C have low fecal concentrations of bile acids ^22^. It is unclear to what extent these findings represent a primary disturbance (that is, due to reduced secretion or increased reabsorption or both) or are secondary to slow colon transit. Nonetheless, because bile acids stimulate colonic secretion of water and high-amplitude-propagated contractions ^23, 24^, it is hypothesized that a paucity of colonic bile acids may cause constipation. Indeed, the corollary is also true: excess bile acid administered orally ^24^ or modification of the enterohepatic recycling pathway ^25^ can exert a laxative effect.

## Normal and abnormal colonic and anal structure and function

Defecation is an intricate viscerosomatic process ^26^, and the pathophysiology of defecatory disorders is heterogeneous. A clinical history is insufficient for differentiating the subtypes of constipation ^27^. A detailed digital rectal examination ^28^ may suggest the presence of manometric features of a defecatory disorder—positive predictive value (PPV) of 97% and negative predictive value (NPV) of 37%—but is less useful for predicting an abnormal rectal balloon expulsion test (PPV of 33% and NPV of 65%) ^29, 30^. Indeed, agreement among anorectal tests is variable, perhaps partly because they assess different aspects of structure or function or both ^31, 32^. Consequently, the Rome criteria recommend that when standard empiric laxative therapy fails to provide relief for patients with constipation, defecatory disturbances be documented with two tests ^32^. However, where manometry or defecography is not available, a digital rectal examination and balloon expulsion test are sufficient for screening.

A detailed rectoanal manometry and rectal balloon expulsion test can provide information regarding rectoanal neuromuscular functions ^33^. Relative to water-perfused manometry, high-resolution manometry is simpler to perform ^34^. However, in clinical practice, the incremental utility of high-resolution manometry above water-perfused manometry is unclear ^33^.

In contrast to high-resolution anorectal manometry, high-definition anorectal manometry provides a three-dimensional evaluation of pressures within the anal canal. To date, relatively small studies suggest that high-resolution and high-definition anorectal manometry have similar performance characteristics for evaluating anorectal disorders ^35, 36^. In high-definition anorectal manometry, similar to high-resolution anorectal manometry, 70% of healthy individuals exhibit a dyssynergic manometry pattern during simulated evacuation ^36^. More recent studies observed that both high-resolution and high-definition anorectal manometry can identify rectoceles, intra-anal intussusception, and rectal prolapse ^37– 40^. However, whether these techniques will augment or supplant defecography, or fall by the wayside, for diagnosing these conditions has yet to be determined.

Within the colon, high-resolution manometry is better than standard water-perfused manometry for visualizing propagated colonic contractions ^41^. While high-resolution colonic manometry remains primarily a research tool, attempts are being made to incorporate these data into clinical practice. For example, among 18 pediatric patients with slow transit constipation and six children with normal transit constipation, manometric findings were predictive of neuropathy with a sensitivity of 100% and a specificity of 86% ^42^.

Radiological tests are also useful for evaluating anorectal and colonic functions. Barium defecography is used to exclude or diagnose rectoceles and pelvic organ prolapse causing obstructed defecation. Magnetic resonance imaging (MRI) defecography offers a radiation-free alternative. Furthermore, it provides a comprehensive evaluation of pelvic organ structure and function during defecation without the need to instill radiopaque material into the small bowel, bladder, or vagina. With the exception of internal intussusception ^43^ and retentive rectoceles ^44, 45^, which are less frequently identified during MRI defecography, barium and MRI defecography have similar performance characteristics.

MRI has been used as a research tool to evaluate colonic motor function in constipation and IBS ^46– 48^. Inoh *et al*. evaluated the relationship between colonic diameter and gastrointestinal symptoms in 20 patients with self-reported chronic constipation by using abdominal MRI ^49^. Ascending colon diameter correlated with a sense of incomplete evacuation, and rectal diameter correlated with constipation scores ^49^. However, although the sum of all the segmental diameters (cecum, ascending, transverse, descending, sigmoid colon, and rectum) correlated with an increasing severity of constipation, the sum of certain segmental diameters also positively correlated with diarrhea. Furthermore, no statistical adjustment appears to have been made for the multiple comparisons performed in this study. Although the hypothesis is interesting, further studies are required to understand the relationship among colonic fecal volume, colonic diameter, and bowel function. Park *et al*. demonstrated that rectal gas volume was a marker of defecatory disorders; at a specificity of 90%, a rectal gas volume of 30 mL had a PPV of 77.3% for an evacuation disorder ^50, 51^.

## Development of novel therapeutic agents for the treatment of constipation

A few medications that selectively target intestinal secretion or motility are available. Serotonin receptor agonists have been used to accelerate intestinal transit. Activation or inhibition of intestinal ionic transporters can increase luminal fluid content and accelerates the rate of colonic transit. Inhibition of ileal bile acid transporters exposes the colon to a greater concentration of these ionic detergents, resulting in the secretion of water into the colonic lumen and accelerated colonic transit.

### Selective 5-HT(4) receptor agonists: prucalopride and velusetrag

Cisapride and tegaserod, the initial 5-HT(4) receptor agonists used to treat functional bowel disorders ^52, 53^, were withdrawn from the market because of cardiovascular events ^54– 56^. By targeting differing pharmacophores, with greater receptor selectivity, novel 5-HT(4) receptor agonists avoid this pro-arrhythmic risk ^57, 58^.

Prucalopride is a selective, high-affinity, 5-HT(4) receptor agonist with prokinetic gastrointestinal activity ^59^. Prucalopride accelerated colonic transit in healthy individuals ^60, 61^ and gastric, small bowel, and colonic transit in constipated patients ^62^. The initial phase 3, double-blind, parallel-group, placebo-controlled trials demonstrated that prucalopride was substantially more efficacious than placebo for increasing the number of spontaneous complete bowel movements by one per week (47% versus 26%, *p* <0.001) and promoting more than three complete spontaneous bowel motions (CSBMs) per week (31% versus 12%, *p* <0.001). Patients reported less-severe symptoms and improved satisfaction with their bowel function ^63^. Subsequent findings include improved constipation-related quality of life ^64^, satisfaction with prucalopride in patients who were dissatisfied with previous laxative treatments ^65, 66^, and efficacy for treating constipation in men ^67^, elderly patients ^68^, and patients with chronic intestinal pseudo-obstruction ^69^, opioid-induced constipation ^70^, or spinal cord injury ^71^. Moreover, prucalopride remains efficacious after 18 months of therapy ^72^. Only one study, a double-blind, placebo-controlled trial over 24 weeks, demonstrated no benefit above placebo ^73^. Even in older patients, the risk of cardiac events, including QT prolongation, is not increased ^74, 75^. Only 5% of patients discontinue the medication because of adverse effects (for example, abdominal pain, nausea, diarrhea, or headache) ^72^. Prucalopride is approved by the European Medicines Agency (EMA), but not by the US Food and Drug Administration (FDA), for the treatment of constipation.

Velusetrag (TD-5108), a newer selective 5-HT(4) receptor agonist, accelerates colonic and gastric transit ^76, 77^. A phase 2 trial of about 400 patients demonstrated a significant increase above placebo in the number of spontaneous bowel motions (about 3.5 versus 1.4, *p* <0.001) and CSBMs per week (about 2 versus 0.6, *p* <0.001) for all doses of velusetrag ^78^. A phase 2 trial of naronapride (ATI-7505) demonstrated beneficial physiological and clinical effects ^79^. These studies with velusetrag and ATI-7505 were published almost a decade ago. In November 2016, after a considerable delay, the FDA recommended that efficacy and cardiovascular safety of naronapride be evaluated in two additional phase 3 studies with 1,000 patients each ^80^. However, no phase 3 trials of velusetrag for constipation are currently registered on ClinicalTrials.gov. Another highly selective 5-HT(4) receptor agonist, YH12852, accelerated upper and lower intestinal transit in animal models ^81^. Human studies are awaited.

### Intestinal chloride channel activators: lubiprostone, linaclotide, and plecanatide

The secretion of ions, and thereby fluid, into the intestinal lumen through ion channels can be pharmacologically driven by lubiprostone, linaclotide, and plecanatide. Activation of the cystic fibrosis transmembrane conductance regulator (CFTR) on the apical surface of enterocytes results in chloride secretion into the intestinal lumen, which is followed by a net secretion of sodium and subsequently water ^82^.

Lubiprostone is a prostaglandin E analog that activates apical type 2 chloride channels, prostaglandin EP receptors, and the apical CFTR ^83^. In a 4-week randomized parallel-group placebo-controlled phase 3 trial involving 237 patients with chronic constipation, lubiprostone (24 μg daily) was superior to placebo ^84^. Lubiprostone-treated patients experienced more frequent spontaneous bowel motions than those treated with placebo (5.9 versus 4.0, *p* <0.001). Lubiprostone reduced bloating ^85^ but did not affect pain thresholds during colonic distention ^86^. It is efficacious for treating constipation associated with cystic fibrosis ^83^, diabetes ^87^, and opioids ^88^. In general, lubiprostone is well tolerated. However, nausea (20%), diarrhea (10%), abdominal distension (7%), headache (7%), and abdominal pain (5%) are reported frequently ^85^.

Linaclotide and plecanatide are uroguanylin analogs that activate cell-surface guanylate cyclase-C receptors on enterocytes, inducing translocation of the CFTR to the apical surface of the cell. Initial studies of linaclotide demonstrated a dose-dependent increase in colonic transit with an associated increase in bowel motion frequency and consistency and reduced straining scores in patients with IBS-C ^89^ and chronic constipation ^90^. Larger studies confirmed these findings, and only 4% of patients stopped the medication because of adverse side effects ^91– 93^. Linaclotide 145 μg and 290 μg increased the mean number of CSBMs per week to about 2.5 versus 0.9 with placebo ( *p* <0.001) ^92^. In rodent models of visceral pain, linaclotide reduced visceral sensitivity ^94^. Perhaps this explains, at least in part, why linaclotide reduced abdominal pain and improved bowel motion frequency and consistency in patients with IBS-C ^95^. These benefits appear to persist with longer-term administration ^96^.

In 2017, plecanatide, which works via mechanisms similar to those of linaclotide, was approved by the FDA for treating chronic idiopathic constipation ^97^ based on a phase 3, multi-center, double-blind, placebo-controlled study of 1,394 patients ^98^. Plecanatide increased the weekly number of CSBMs (about 2.2 versus 1.2, *p* <0.001) and spontaneous bowel motions (about 3.1 versus 1.3, *p* <0.001) per week above those seen with placebo during a 12-week study. Adverse effects (most commonly diarrhea) occurred in about 6% of patients taking plecanatide and 1% of patients receiving placebo. Similar findings were reported elsewhere ^99^. An open-label follow-up study of 2,370 patients who had been enrolled in phase 2b or phase 3 studies demonstrated that 82% had completed or were still receiving the study drug ^100^. These patients reported a median satisfaction score for treatment of 4.0 (quite satisfied) and were “quite likely” to continue the medication. Animal models suggest that plecanatide, similar to linaclotide, also reduces visceral sensitivity ^101^, and phase 3 trials in patients with IBS-C have demonstrated a significant improvement in bowel motion frequency, stool consistency, and abdominal pain above that seen with placebo ^102^. Sustained response with the 6 mg dose of plecanatide was seen in 30% of patients in the first study (placebo response 18%, *p* <0.001) and 24% in the second trial (14% placebo response, *p* <0.001) ^102^. Additional CFTR activators (CFTRact-J027 and its derivatives) have demonstrated efficacy for treating constipation in mouse models ^103, 104^.

### Modifiers of bile acid recycling and synthesis: elobixibat and NGM282

Elobixibat (A3309) is the first-in-class ileal bile acid transporter inhibitor ^105^. Inhibiting the absorption of bile acids from the ileum exposes the colonic mucosa to a higher concentration of these ionic detergents. This accelerates colonic transit, increases stool frequency, and potentially relieves the symptoms of constipation ^23, 24, 106^. In a randomized phase 2b trial, 190 patients with chronic constipation received 5, 10, or 15 mg of elobixibat or placebo once daily for 8 weeks. The times to the first spontaneous bowel motion and CSBM were significantly shortened in the 10 and 15 mg groups. Stool frequency and constipation-related symptoms were significantly improved ^107^. A phase 3 trial confirmed the efficacy of elobixibat for the treatment of chronic constipation but demonstrated that adverse drug reactions occur in up to half of patients ^108^. These are usually mild abdominal pain or diarrhea. Though approved for clinical use in Japan, elobixibat is not currently approved for use by the EMA or the FDA.

In patients with functional constipation, the fibroblast growth factor 19 analog NGM282 accelerated gastric and colonic transit, resulting in an increased number of bowel movements, looser stool form, and increased ease of stool passage ^25^. The rationale for this study was the observation that NGM282 induced diarrhea in phase 2 trials relating to type 2 diabetes, primary biliary cholangitis, and non-alcoholic steatohepatitis. However, NGM282 is a potent inhibitor of bile acid synthesis, and in this study, in contrast to the effect of elobixibat, bile acid concentration in stool was reduced. Further physiological studies are needed to elucidate how NGM282 exerts its effects.

### Sodium/hydrogen exchanger inhibitors: tenapanor

Tenapanor (AZD1722) is a first-in-class, minimally absorbed, small-molecule inhibitor of the gastrointestinal sodium/hydrogen exchanger NHE3. It inhibits the absorption of dietary sodium and phosphate, which increases intestinal fluid volume and transit ^109^. The effect is more pronounced when tenapanor is administered pre-meal ^110^. A phase 2 randomized placebo-controlled trial of 356 patients—87% women, mean ± standard deviation (SD) of 46 ± 13 years—with IBS-C demonstrated a CSBM responder rate of 61% with tenapanor 50 mg twice a day as compared with 34% in the placebo group ( *p* <0.001). Additionally, abdominal pain was significantly reduced in the tenapanor group ^111^. The most common adverse events were diarrhea, headache, nausea, urinary tract infection, and abdominal pain. Diarrhea occurred in 9% of patients and resulted in medication cessation in 3%. Of note, data regarding the effect of tenapanor on hyperphosphatemia in patients with chronic kidney disease, which encompassed an older patient cohort (mean ± SD of 59 ± 14 years), demonstrated a similar side effect profile ^112^. Thereafter, two phase 3 trials in IBS-C, each with about 600 patients treated for 12 and 26 weeks, have been reported. In the first phase 3 trial, tenapanor met its primary endpoint (combined pain and stool pattern responder rate of 27% versus 19% with placebo, *p* = 0.02) ^113^. Significance was met for the secondary endpoint of abdominal pain relief but not for the CSBM endpoint. In the second study, tenapanor met all primary and secondary endpoints ^114^; the company plans to submit an application to the FDA in the second half of 2018. Tenapanor is not licensed by the EMA.

### New but not necessarily better

Although these new agents are efficacious, only two studies have directly compared the clinical efficacy of newer and older laxatives. The first study compared polyethylene glycol with tegaserod, which has since been withdrawn from the market, in a randomized open-label, parallel-group study of 237 patients. Polyethylene glycol was better for improving symptoms of constipation. In the second, a randomized, double-blind, double-dummy study of 240 patients with chronic constipation, polyethylene glycol with electrolytes was compared with prucalopride ^115^. Polyethylene glycol with electrolytes was non-inferior to prucalopride for promoting more than three CSBMs per week and was better tolerated.

Indeed, a network meta-analysis comparing prucalopride, lubiprostone, linaclotide, tegaserod, velusetrag, elobixibat, bisacodyl, and sodium picosulfate was undertaken, albeit with limited data for some medications, and observed that these drugs were of comparable efficacy for the endpoints of at least three CSBMs per week or an increase over baseline of at least one CSBM per week. Bisacodyl was superior to the other agents for inducing a greater change from baseline in the number of spontaneous bowel motions per week ^116^.

Furthermore, the newer laxatives are much more expensive than the older, over-the-counter agents. In the United States, a 30-day supply of lubiprostone, linaclotide, or plecanatide costs about $450. By comparison, a 30-day supply of psyllium, polyethylene glycol, bisacodyl, or senna costs less than $10, whereas lactulose costs less than $15.

## Optimizing therapy and the potential role for individualized treatment of constipation

Consensus guidelines recommend that pelvic floor biofeedback therapy, not laxatives, is the cornerstone for managing defecatory disorders ^8^. However, none of the pharmacological studies described above rigorously evaluated anorectal function and excluded patients with defecatory disorders. Therefore, the relative efficacy of these drugs in patients with isolated normal or slow transit constipation is unknown.

Where constipation does not respond to empiric therapy and a defecatory disorder has been excluded, investigation could guide tailored therapy. For example, the 5-HT(4) receptor agonist prucalopride might be a preferential choice for patients with diffusely slow intestinal transit. Ileal bile acid transporter inhibitors could be of benefit for constipated patients with a deficiency of bile acids reaching the colon. A single case report observed that mexiletine normalized bowel functions in a patient with IBS-C and a mutation in the *SCN5A* gene, which encodes the alpha-subunit of the voltage-gated sodium channel Na _V_1.5 ^20^.

Future studies should evaluate the efficacy of novel and standard laxatives in patients with defined normal or slow transit constipation and the efficacy of targeted therapy (for example, with ileal bile acid transporter inhibitors in constipated patients with a deficiency of bile acids reaching the colon).

## Summary and conclusions

Constipation is common, but the underlying pathophysiology remains unclear in many cases. Many patients can be effectively and inexpensively treated with simple laxatives. Newer intestinal secretagogues and promotility agents are more expensive and should be considered in patients who do not respond to simple laxatives. A few new agents are being evaluated in clinical trials. There is a critical need to compare the efficacy of these newer agents relative to established laxatives and also clarify their efficacy in the subtypes of constipation (that is, normal transit, slow transit, and defecatory disorders).
